# Major antigen and paramyosin proteins as candidate biomarkers for serodiagnosis of canine infection by zoonotic *Onchocerca lupi*

**DOI:** 10.1371/journal.pntd.0009027

**Published:** 2021-02-10

**Authors:** Maria Stefania Latrofa, Giuseppe Palmisano, Giada Annoscia, Ciro Leonardo Pierri, Ramaswamy Chandrashekar, Domenico Otranto

**Affiliations:** 1 Department of Veterinary Medicine, University of Bari, Bari, Italy; 2 Department of Parasitology, ICB, Glycoproteomics Laboratory, University of São Paulo, Brazil; 3 Department of Biosciences, Biotechnologies and Biopharmaceutics, Laboratory of Biochemistry and Molecular Biology, University of Bari, Bari, Italy; 4 IDEXX Laboratories, Inc., 1 IDEXX Drive, Westbrook, Maine, United States of America; 5 Department of Pathobiology, Faculty of Veterinary Science, Bu-Ali Sina University, Felestin Sq., Hamedan, Iran; University of Utah, UNITED STATES

## Abstract

*Onchocerca lupi* (Spirurida: Onchocercidae) is a filarial worm parasitizing domestic carnivores and humans. Adult nematodes usually localize beneath in the sclera or in the ocular retrobulbar of infected animals, whilst microfilariae are found in the skin. Therefore, diagnosis of *O*. *lupi* is achieved by microscopic and/or molecular detection of microfilariae from skin biopsy and/or surgical removal of adults from ocular tissues of infected hosts. An urgent non-invasive diagnostic tool for the diagnosis of *O*. *lupi* in dog is mandatory. In this study, an immunoproteomic analyses was performed using a combination of immunoblotting and mass spectrometry techniques. *Onchocerca lupi* major antigen (*Ol*-MJA) and paramyosin (*Ol*-PARA) proteins were identified as potential biomarkers for serodiagnosis. Linear epitopes were herein scanned for both proteins using high-density peptide microarray. Sera collected from dog infected with *O*. *lupi* and healthy animal controls led to the identification of 11 immunodominant antigenic peptides (n = 7 for *Ol*-MJA; n = 4 for *Ol*-PARA). These peptides were validated using sera of dogs uniquely infected with the most important filarioids infesting dogs either zoonotic (*Dirofilaria repens*, *Dirofilaria immitis*) or not (*Acanthocheilonema reconditum* and *Cercopithifilaria bainae*). Overall, six antigenic peptides, three for *Ol*-MJA and for *Ol*-PARA, respectively, were selected as potential antigens for the serological detection of canine *O*. *lupi* infection. The molecular and proteomic dataset herein reported should provide a useful resource for studies on *O*. *lupi* toward supporting the development of new interventions (drugs, vaccines and diagnostics) against canine onchocercosis.

## Author summary

The diagnosis of *Onchocerca lupi* (Spirurida: Onchocercidae), a zoonotic nematode of domestic animals, is currently based on microscopic examination of skin snip sediments and on the identification of adults embedded in ocular nodules or by molecular assays. An urgent non-invasive diagnostic tool for the diagnosis of *O*. *lupi* in dogs is mandatory. In this context, combined immunoblotting and mass spectrometry-based analyses have been performed to identify two proteins, major antigen and paramyosin, of *O*. *lupi*. Peptides herein identified represent suitable candidate biomarker for the development of a specific diagnostic test for canine onchocercosis. An accurate, minimally invasive diagnostic method could prove useful for the control of the canine diseases, for establishing large sero-surveys, for mapping the distribution of the infection in endemic areas as well as in areas where information on the disease is not available and for the reduction of risks for human infection.

## Introduction

Nematodes of the family Onchocercidae are common parasites of wild and domestic ungulates, carnivores and humans [1–3]. Unlike the most studied species *Onchocerca volvulus*, the causative agent of the river blindness, which has a large-scale public health concern [4], data on the epidemiology and zoonotic potential of *Onchocerca lupi*, the agent of canine ocular onchocercosis, are still scant [5–9]. *Onchocerca lupi* was firstly detected from a Caucasian wolf (*Canis lupus*) in Georgia [10] and subsequently diagnosed in domestic animals (i.e., dogs and cats) from European countries (i.e., Hungary, Greece, Germany and Portugal) and USA [9, 11–17]. The zoonotic role of *O*. *lupi* was reported for the first time in 2011 [7] and, since then on, up to 18 patients have been diagnosed positive for this parasite, worldwide (i.e., Germany, Tunisia, Hungary, Greece, Turkey, Iran and USA) [18–20]. The diagnosis of canine onchocercosis is achieved by detection and identification of microfilariae (mfs) in small skin biopsies, an invasive procedure not often accepted by dog owners, mainly in absence of typical clinical lesions of the infection [21, 22], and or based on the presence of ocular nodules on the eyelids, conjunctiva, and sclera of dogs [23, 24]. However, the results of these procedures may be (false-) negative in the case of infections with immature or not reproducing worms and according to the day-time of the sampling, considering the circadian rhythm of mfs [8]. Even if, PCR-based DNA assays have been developed [7, 25] and preliminary investigations on serology have been attempted [26, 27], a serological, non-invasive diagnosis is still missing. Although, the preliminary immunological properties of paramyosin protein of *O*. *lupi* (*Ol*-PARA) has been previously evaluated, no immunoreactive peptides have been identified [27]. Therefore, the aim of this study was to use an immunoproteomic approach combining immunoblotting and mass spectrometry-based analyses to identify novel antigens that might lead to improve diagnostic tests for canine onchocercosis. The molecular and proteomic dataset herein reported should establish large sero-surveys for mapping the distribution of the infection in endemic areas as well as in areas where information on the disease is not available.

## Materials and methods

### Ethics statement

The study was conducted according to the Guideline on Good Clinical Practices (The European Agency for the Evaluation of Medicinal Products, Veterinary Medicines and Information Technology Unit, VICH Topic GL9; www.emea.eu.int/pdfs/vet/vich/059598en.pdf) and procedures were approved by the ethical commission at the University of Évora (identification number: AE02Fila2013), complying with Portuguese legislation for the protection of animals (Decree-Law no. 113/2013). An owner consent agreement was obtained before sample collection.

**Fig 1:**
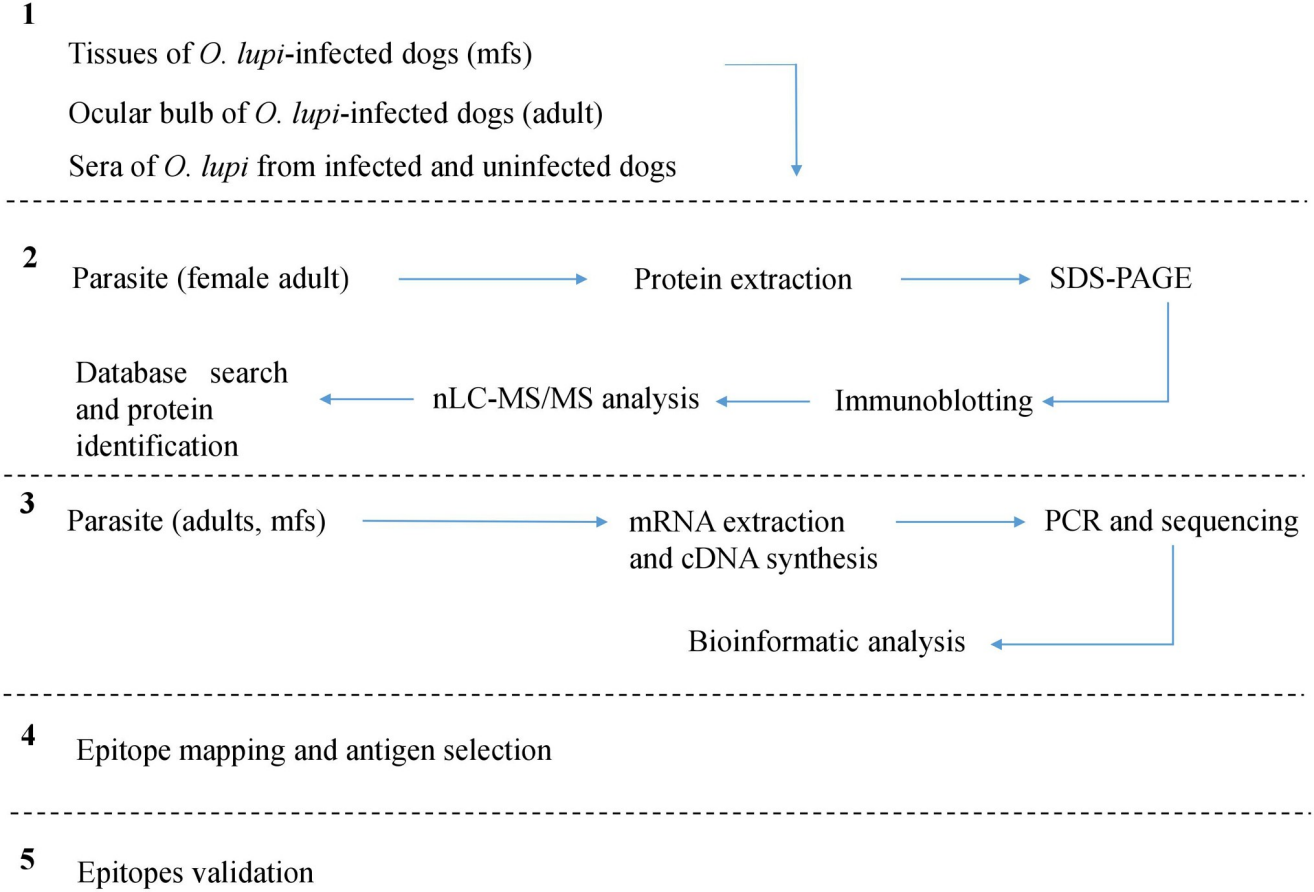
Experimental workflow for the identification of novel *Onchocerca lupi* antigens. Five steps were performed: 1) Parasites (adults and mfs) and sera collection; 2) Immunoproteomic approach. Adult female parasite was lysed and proteins extracted before separating them in a SDS-PAGE were blotted into a PVDF membrane and probed using sera collected from dogs infected with *O*. *lupi*. Bands at approximately 100 and 200kDa molecular weight were identified and analysed by LC-MS/MS. Major antigen and paramyosin proteins were identified. 3) Identification of *Ol*-Mja nucleotide sequence. cDNA of Major antigen protein gene from different life stages of parasite were obtained by RT-PCR and analysed by bioinformatics tools. 4) Immunogenic linear epitope scanning of *Ol*-MJA and *Ol*-PARA proteins was performed by high density-peptide array screening using sera collected from uninfected dog, *O*. *lupi*-infected dog and dogs infected with other helminths. 5) Epitopes validation was performed by incubation of epitopes with serum samples of *O*. *lupi*, *Dirofilaria repens*, *Cercopithifilaria bainae*, *Dirofilaria immitis* and *Acanthocheilonema reconditum* positive dogs and serum from negative dog.

Flowchart description of the experimental procedure and computational analysis of candidate serum-diagnostic proteins have been reported in Fig 1.

### Samples collection

*Onchocerca lupi* female (n = 3) and male (n = 2) specimens have been isolated from the eye of an infected dog (3-year-old, male) that died accidentally in Algarve region (southern Portugal) where *O*. *lupi* is known to occur [16]. Eggs were collected by cutting the uterus of female, whilst mfs by sedimentation from the skin of the dorsal region of the dog (see below). All samples were preserved in RNAlater (Life Technologies, California, USA) and stored at -80°C until used.

Skin samples were collected using a disposable punch over an area of ≈0.4 × 0.5 cm from the interscapular region of the dog, soaked in saline solution for 12 h at room temperature. Sediments (20 μl) were individually observed under light microscopy and mfs were identified according to morphological keys [16].

Serum samples, one for each pathogen, were obtained from *O*. *lupi* positive dog and from animals solely infected with *Cercopithifilaria bainae*, *Acanthocheilonema reconditum*, *Dirofilaria repens* and *Dirofilaria immitis*, sourced from previous researches [7, 28]. As negative control serum sample collected from pathogen-free dog [29] was included in the study.

### SDS-PAGE and Western blotting

Proteins were extracted from an adult female of *O*. *lupi* using the TriPure (Roche Molecular Biochemicals, Basel, Switzerland) protocol as described below and quantified (0.55 μg/μl) using the QUBIT Protein Assay kit (Foster City, California, USA) according to the manufacturer instructions and subjected to SDS-PAGE and Western Blotting as previously described [30, 31]. Total proteins (24 μl) were added to the same volume of 1x Sodium Dodecyl Sulphate (SDS) sample buffer and 1 μl of β-mercaptoethanol, heated to 100°C for 10 min and microcentrifuge at 12000x g for 5 min. Each well of the two 7.5% SDS polyacrylamide gels (SDS-PAGE) were loaded with 3 μl of *O*. *lupi* protein and with 5 μl of pre-stained standard (PageRuler Plus Prestained Protein Ladder, Thermo Scientific Waltham, Massachusetts, USA) according to the manufacturer instructions. Both gels were run at 200V for ~45min. Proteins from the gel were transferred to polyvinylidene fluoride (PVDF) membrane (Hercules, California, USA) in a semi-dry blotting cell (0.5A/45min) using the BioRad TurboBlot (Hercules, California, USA). For immunostaining, each blot was incubated at 4°C overnight (O/N), in blocking solution (PBS/0.1%Tween-20/5% skim milk powder). Following blocking, membranes were incubated at 4°C with serum from an uninfected dog (negative control) or those infected with *O*. *lupi*, *D*. *immitis*, *D*. *repens*, *A*. *reconditum* and *C*. *bainae* (1:1000 dilution), respectively. Dog antibodies were detected using rabbit anti-dog IgG horseradish peroxidase conjugate (1:3000; Sigma Adrich, Missouri, USA) and detected using the FemtoChromo kit (G Biosciences, Missouri, USA). The second SDS-PAGE was stained with colloidal blue and putative bands at the same molecular weight as the blotting, were cut and examined by nLC-MS/MS analysis from Cogentech Proteomics/MS facility (Milan, Italy).

The SDS-PAGE of the *O*. *lupi* female protein in reducing conditions was included as S1 Fig.

### In gel protein digestion

The gel slices were washed sequentially three times for 15 min with acetonitrile (ACN) 50% and 25 mM ammonium bicarbonate (NH_4_HCO_3_), dehydrated with ACN 100% for 5 min followed by vacuum centrifugation. Disulfide bridges were reduced using 10 mM DTT in 100 mM NH_4_HCO_3_ for 1 h at 56°C. The reduced cysteines were alkylated with 55 mM iodoacetamide in 100 mM NH_4_HCO_3_ for 45 min in the dark, washed twice for 15 min with 100 mM NH_4_HCO_3_. Gel slices were dehydrated with ACN 100% for 5 min followed by vacuum centrifugation and the digestion buffer containing Sequencing grade Trypsin (Promega, Madison, WI, USA) in 20 mM ammonium bicarbonate, incubated for 1 h at room temperature and then overnight at 37°C. The supernatant was transferred to a low protein binding tube and tryptic peptides were extracted from the gel slices using sequentially ACN 50%, trifluoroacetic acid (TFA) 5% for 30 min. The peptides were desalted using Stage Tips with C18 disks (Sigma Aldrich, St. Louis, Missouri, USA).

### Liquid chromatography–tandem MS (LC–MS/MS) analysis

Two bands identified by SDS-PAGE and Western blotting were cut from gel and trypsinized as previously described [32]. Peptides were desalted as described by [33], dried in a Speed-Vac and re-suspended in 10 μl of solvent A (2% ACN, 0.1% formic acid). Of them, 3 μl were injected on a quadrupole Orbitrap Q-Exactive mass spectrometer (Thermo Scientific) coupled with an UHPLC Easy-nLC 1000 (Thermo Scientific) with a 25 cm fused-silica emitter of 75 μm inner diameter. Columns were packed in-house with ReproSil-Pur C18-AQ beads (Dr. Maisch Gmbh, Ammerbuch, Germany), 1.9 μm of diameter, using a high-pressure bomb loader (Proxeon, Odense, Denmark). Peptides separation was achieved with a linear gradient from 95% solvent A (2% ACN, 0.1% formic acid) to 40% solvent B (80% acetonitrile, 0.1% formic acid) over 30 min and from 40% to 100% solvent B in 2 min at a constant flow rate of 0.25 μl/min, with a single run time of 33. MS data were acquired using a data-dependent top 12 method, the survey full scan MS spectra (300–1750 Th) were acquired in the Orbitrap with 70000 resolution, AGC target 1e6, IT 120 ms. For HCD spectra resolution was set to 35000, AGC target 1e5, IT 120 ms; normalized collision energy 25% and isolation width of 3.0 m/z. The mass spectrometry proteomics data have been deposited to the ProteomeXchange Consortium via the PRIDE [34] partner repository with the dataset identifier PXD016311.

### Protein identification

For protein identification the raw data were processed using Proteome Discoverer (version 1.4.0.288, Thermo Fischer Scientific, Waltham, Massachusetts, USA). MS^2^ spectra were searched with Mascot engine against uniprot_swsprot_all_20170110 database (553231 entries), with the following parameters: enzyme Trypsin, maximum missed cleavage 2, fixed modification carbamidomethylation (C), variable modification oxidation (M) and protein N-terminal acetylation, peptide tolerance 10 ppm, MS/MS tolerance 20 mmu. Peptide Spectral Matches (PSM) was filtered using Percolator based on q-values at a 0.01 FDR (high confidence). Proteins were considered identified with 2 unique high confident peptides [35]. Scaffold (version Scaffold 4.3.4, Proteome Software Inc., Portland, OR) was used to validate MS/MS based peptide and protein identification. Peptide identification was established with a greater than 95% probability by the Peptide Prophet algorithm [36] with Scaffold delta-mass correction. Protein identification was accepted with a greater than 99% probability and contained at least 2 peptides. Protein probabilities were assigned by the Protein Prophet algorithm [37]. Proteins that contained similar peptides and could not be differentiated based on MS/MS analysis alone were grouped to satisfy the principles of parsimony. Proteins sharing significant peptide evidence were grouped into clusters.

### RNA isolation and cDNA synthesis

Total RNA was isolated from adults (male and female), mfs and eggs of *O*. *lupi*, following homogenization using a pestle and 400–600 μm glass beads (Sigma-Aldrich, Missouri, USA) employing the TriPure isolation reagent according to the manufacturer's instructions (Roche Molecular Biochemicals, Basel, Switzerland). RNase inhibitor (RNasin, Promega, Wisconsin, USA) was added to total RNA before quantification and storage at -80°C. Due to the tiny amount of RNA able to be extracted from these nematodes, no DNase treatment was performed. Nucleic acid was quantified using the QUBIT RNA Assay kit (Foster City, California, USA) according to the manufacture instructions. First strand cDNA synthesis was performed using the Super- Script Reverse Transcriptase II kit (Invitrogen, California, USA), 0.5 g oligo dT primer (n = 12–18 primer, Promega, Wisconsin, USA) and 100 ng of total RNA from adult male and female, mfs and eggs according to the manufacturer instructions. Each completed reaction was diluted to 250 ng/l with TE (10 mM Tris-HCl, 1 mM EDTA, pH 8.0) before use in reverse transcription PCR.

### PCR amplification, sequencing and nucleotide assembly of *Onchocerca lupi* Major antigen

Amplification of the *O*. *lupi*-Major antigen protein (*Ol*-Mja) gene (~6300bp) was performed using 250 ng of cDNA of female, male, pool of mfs and eggs, using Phusion High-Fidelity DNA polymerase (New England Biolabs, Thermo Scientific, Ipswich, Massachusetts, USA) and seven primer pair sets (S1 Table). Primers were designed on *O*. *volvulus* Major antigen (ovt1-gene) nucleotide sequence (Accession U12681.1_OVT1) using Primer3web version 4.1.0 [38]. PCR reactions consisted of 1×GC reaction buffer, 2.5 pmol of each primer, 0.6 mM of dNTPs and 1 U of DNA polymerase in a volume of 20 μl. Cycling conditions were reported in S1 Table. Products were resolved on 1% agarose stained with 0.5× GelRed (Biotium, California, USA) and visualized on a GelLogic 100 gel documentation system (Kodak, New York, USA). Amplicon sequencing was achieved using the same PCR primer pairs for each fragment, the Big Dye Terminator v.3.1 chemistry and the 3130 genetic analyzer (Applied Biosystems, California, USA). Whole *Ol*-Mja nucleotide sequence was assembled from contiguous sequences (contigs) using Geneious Pro (Version 6.06, Auckland, New Zealand), mapping on to the reference sequence of *O*. *volvulus* ovt1-gene. The *Ol*-Mja nucleotide sequence was conceptually translated into protein (*Ol*-MJA) using the standard code by MEGA6 software [39]. The open-reading frame and codon usage profiles of protein-coding genes were analyzed by the Open Reading Frame Finder (http://www.ncbi.nlm.nih.gov/gorf/gorf.html) using the standard code.

### Bioinformatics and phylogenetic analyses

The identity of the full-length nucleotide sequences of *Ol*-Mja from adults, mfs and eggs was confirmed by BLASTn queries from the NR database at NCBI (https://blast.ncbi.nlm.nih.gov/Blast.cgi?PROGRAM=blastn&PAGE_TYPE=BlastSearch&LINK_LOC=blasthome) [40]. Protein sequences of *Ol*-MJA and of *Ol*-PARA were used as query to search for those of Onchocercidae (taxid:6296), Nematoda (taxid: 6231), Metazoan (taxid:33208), Fungi (taxid:4751), Plants (taxid:3193) and Bacteria (taxid:2) from the NR protein database using BLASTp (https://blast.ncbi.nlm.nih.gov/Blast.cgi?PAGE=Proteins) [41]. Protein sequences showing E-value lower than 5*10^−178^, a query coverage greater than 85% and a percentage of identical residues greater than 30% were used for the multiple sequence alignment (MSA) analysis. The MSA of sequences was built by using ClustalW implemented in the sequence editor package Jalview [42] both for Major antigen (MJA) and Paramyosin homologous sequences.

The evolutionary history of homologues MJA sequences was inferred using the Maximum Likelihood (ML) method based on the Jones-Taylor-Thornton (JTT) matrix-based model [43] and a discrete Gamma distribution (+G), selected by best-fit model [44]. Evolutionary analysis was tested with 100 bootstrap replications, using MEGA6 software [39]. The corresponding amino acid sequences from *Acanthocheilonema viteae* and *Wuchereria bancrofti* and *Caenorhabditis elegans* (Accession numbers: VBB26490.1, VDM19311.1, NP4948193.1) were used as outgroups.

### Prediction of signal peptides and protein 3D comparative modelling

The predicted *Ol*-MJA protein was subjected to additional analyses for the presence of signal peptides (http://www.cbs.dtu.dk/services/SignalP/) [45–47], trans-membrane domains and classical secretion peptides using TMHMM Server v. 2.0 and SignalP 4.1 (https://www.expasy.org/) [45] and for the detection of conserved protein domains (ProSite; www.expasy.ch/tools/scnpsit1.html). Potential B-cell epitopes of *Ol*-MJA were predicted using the BepiPred-2.0 (http://www.cbs.dtu.dk/services/BepiPred/). Conserved domain scan was performed using the CD-Search [48] (https://www.ncbi.nlm.nih.gov/Structure/cdd/) [49].

The pGenThreader and I-Tasser webservices were used for identifying putative crystallized structures to be used as protein templates for attempting to build a 3D multi-template comparative model for *Ol*-MJA and *Ol*-PARA proteins, by using Modeller, according to protocols previously described [50].

### Epitopes identification and validation using amino acid slide scan

Peptide array synthesis and binding detection were performed by PEPperPRINT GmbH (PEPperCHIP1 Platform Technology, Heidelberg, Germany). The *Ol*-MJA and *Ol*-PARA proteins sequences were linked and elongated with neutral GSGSGSG linkers at the C- and N-terminus to avoid truncated peptides. The linked and elongated antigen sequences were translated into 15 amino acid peptides with a peptide-peptide overlap of 14 amino acids. The resulting peptide microarrays contained 2,843 different peptides printed in duplicate (5,686 peptide spots), and were framed by additional HA (YPYDVPDYAG, 156 spots) control peptides. Peptide microarray was pre-stained with the secondary antibody in incubation buffer to investigate background interactions with the antigen-derived peptides. Peptide arrays was incubated with 1:1000 serum dilution samples of *O*. *lupi* positive and negative dogs and incubated for 16 h at 4°C and shacked at 140 rpm. A secondary antibody (Goat anti-dog IgG (Fc) DyLight680 (0.4 μg/ml)) was added for 45 min and stained in incubation buffer at room temperature (RT). Mouse monoclonal anti-HA (12CA5) DyLight800 (0.5 μg/ml) was added for 45 min and staining in incubation buffer at RT. Arrays were scanned with LI-COR Odyssey Imaging System and scanning fluorescence signals (offset 0.65 mm, resolution 21 μm, scanning intensities of 7/7 (red = 700 nm/green = 800 nm)) were used to calculate relative intensity compared to the native peptide. Quantification of spot intensities and peptide annotation were done with PepSlide Analyzer. The same peptide microarray layout was used to test serum samples of *D*. *repens* and *C*. *bainae* at dilution of 1:1000 and serum samples of *D*. *immitis* and *A*. *reconditum* at dilution of 1:250.

The comparison of epitopes herein identified with those of other nematodes was assessed using ClustalW implemented in the sequence editor package Jalview (see above).

## Results

### Protein identification of *Onchocerca lupi*

**Fig 2:**
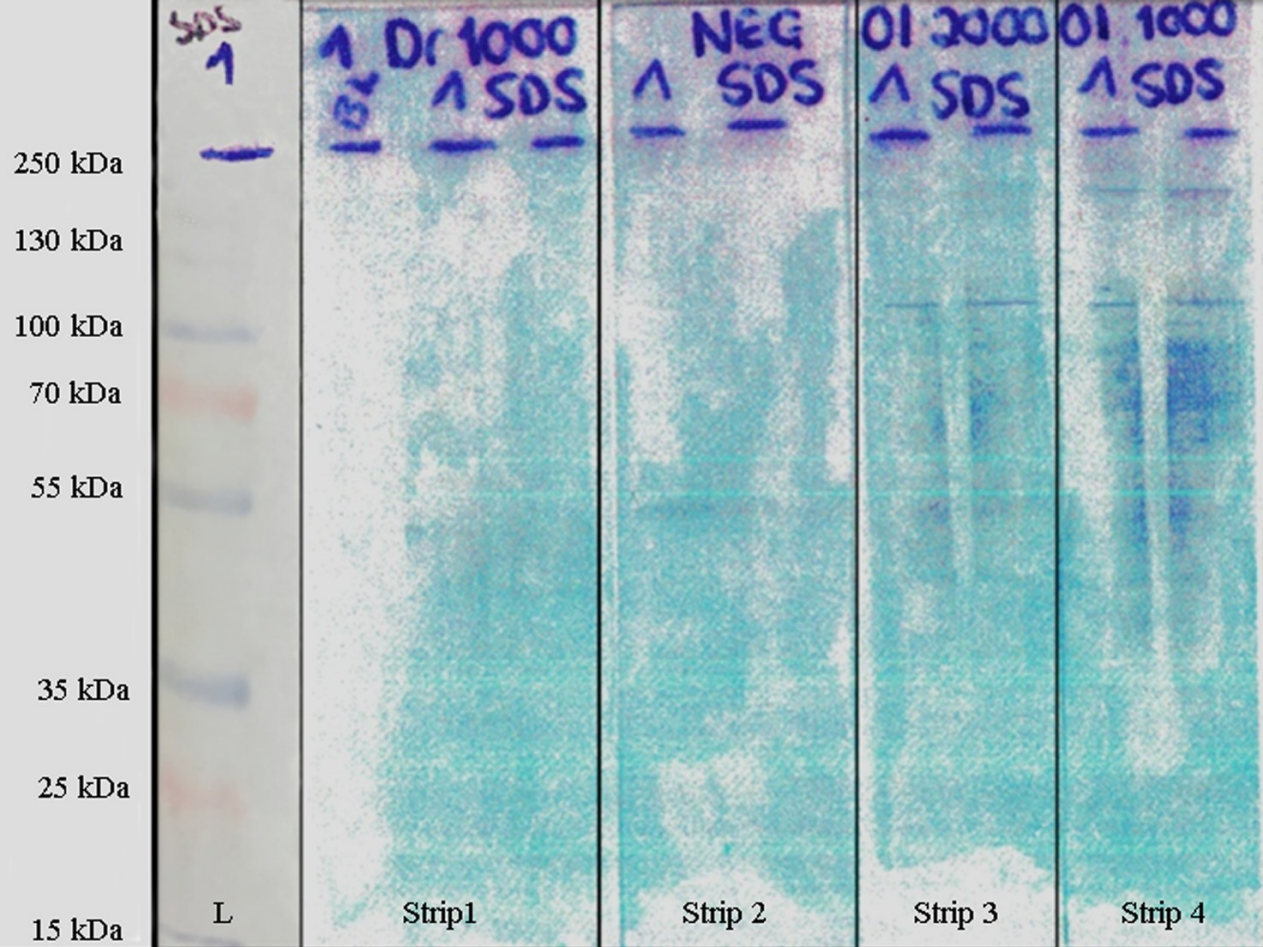
Immunoblotting analysis. Proteins extracted from adult female *Onchocerca lupi* parasite separated by SDS-PAGE and blotted to PVDF membrane. The PVDF membrane strips were incubated with the following serum samples: 1) serum from *Dirofilaria repens* infected dog (diluted 1:1000); 2) serum from pathogen-free dog (negative control); 3) serum from *O*. *lupi* infected dog (diluted 1:2000); 4) serum from *O*. *lupi* infected dog (diluted 1:1000); L = ladder.

PVDF membranes probed with serum samples allowed the identification of a band with a molecular mass of ~200kDa reactive with *O*. *lupi* sample and of ~100kDa reactive with *O*. *lupi* and *D*. *repens* (faint band) samples (Fig 2). The nLC-MS/MS analysis of gel bands resulted in the identification of seven proteins (i.e., Actin 1 and 2, Calponin, Major antigen, Paramyosin, Muscle cell intermediate filament protein and Tropomyosin) with a theoretical molecular weight ranging from 33 to 273KDa, being associated to *O*. *volvulus* species (Table 1). Proteins with a lower molecular weight (i.e., actin and calponin) were detected due to proteoforms or cleaved products. In particular, the analysis of the gel band at ~200kDa identified five proteins (i.e., Actin 2, Calponin, Major antigen, Paramyosin and Muscle cell intermediate filament protein) with a total of 20 and 16 unique tryptic peptides associated to *O*. *volvulus* paramyosin (PARA) and Major antigen (MJA) proteins, respectively (Table 1). All proteins identified by nLC-MS/MS analysis were reported in the S2 Table.

**Table 1:** The nLC-MS/MS analysis. Proteins and number of unique peptides mapped to *Onchocerca volvulus* obtained from the upper (~200kDa) and lower (~100kDa) gel bands. Protein description, organism, Uniprot code (ID), molecular weight (kDa) are reported.

Protein description	Organism	Uniprot ID	Protein molecular mass (kDa)	Unique peptide count	
				Low band (~100kDa)	Up band (~200kDa)
Major antigen	*O*. *volvulus*	ANT1_ONCVO	237	-	16
Paramyosin	*O*. *volvulus*	MYSP_ONCVO	101	-	20
Muscle cell intermediate filament protein	*O*. *volvulus*	OV71_ONCVO	50	-	4
Actin-2	*O*. *volvulus*	ACT2_ONCVO	42	9	7
Actin-1	*O*. *volvulus*	ACT1_ONCVO	42	3	-
Calponin homolog	*O*. *volvulus*	CLPH_ONCVO	42	11	2
Tropomyosin	*O*. *volvulus*	TPM_ONCVO	33	4	-

### Nucleotide and amino acid sequences analyses

**Fig 3:**
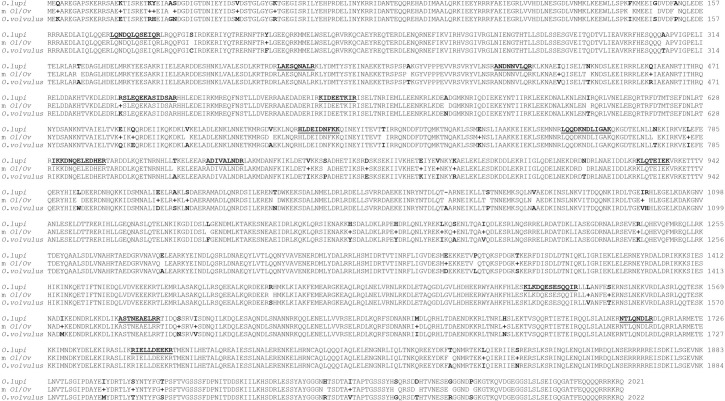
Alignment of Major antigen amino acid sequences of *Onchocerca lupi* and *Onchocerca volvulus*. Amino acids differences are reported in bold. Peptides identified by nLC-MS/MS analysis are underlined and reported in bold. The amino acid substitution matrix (m *Ol/Ov*) is reported in the central line. Identical residues and residues with positive substitution score (+) are indicated.

The 6,163bp of *Ol*-Mja cDNA gene was assembled from a series of contiguous overlapping partial sequences, obtained from each adult (male and female) and mfs without nucleotide (nt) differences among them. No amplification was obtained from cDNA of eggs. BLASTn analysis of *Ol*-Mja sequence revealed the highest nt identity with those of other related filarial nematodes as *O*. *volvulus* (96.47%; 5854/6068 nt; Accession no. U12681.1), *D*. *immitis* (88.9%; 5377/6051 nt; Accession no. JR905253.1) and *Loa loa* (86.24%; 5248/6085 nt; Accession no. XM_020451247.1). Conversely, nt identities up to 97% were observed with short fragments (from 353 to 1945 bp) of other species (Table 2). The *Ol*-Mja sequence contained an open reading frame (ORF) of 100bp of 5’ no translated sequence. The codon for the initiation methionine was the first ATG downstream from the spliced leader, whilst the termination codon was identified at position 6062bp. The *in silico* translated *Ol*-MJA protein consisted of 2021 amino acids (aa) in length (Fig 3), having a theoretical molecular weight of 237,342kDa. The comparison between aa sequences of *Ol*-MJA and that of reference *O*. *volvulus* (P21249.2) resulted in 78 variable sites, of which 44 were similar (chemically-related side chain) and one was a gap (Fig 3). All 16 peptides identified by nLC-MS/MS analysis were found in the *Ol*-MJA sequence (Fig 3).

**Table 2:** Nucleotide identity of *Onchocerca lupi* Major antigen compared with those of other nematode species. Percentage of nucleotide identity, accordingly to sequence coverage, accession number, and protein name are indicated.

Nucleotide				
Species	Protein name	Accession number	Nucleotide coverage	Identity (%)
*Onchocerca volvulus*	Major antigen	U12681.1	5854/6068	96.47
*Dirofilaria immitis*	Unigene9325	JR905253.1	5377/6051	88.9
*Loa loa*	Major antigen	XM_020451247.1	5248/6085	86.24
*Onchocerca gibsoni*	Paramyosin-related protein	U20609.1	1945/2017	96.4
*Onchocerca volvulus*	Myosin-like antigen	M30398.1	1935/2016	96
*Ancylostoma caninum*	AIAC-aaa78e06.g1	EX543135.1	633/673	94.1
*Brugia malayi*	Major antigen	XM_001893962.1	601/726	82.78
*Brugia malayi*	SWYD25CAU10B11SK	AW347993.1	544/650	83.7
*Brugia malayi*	SW3ICA2436SK	AA255396.1	463/552	83.9
*Onchocerca volvulus*	Myosin-like antigen	AH001078.2	442/458	96.5
*Onchocerca volcalus*	Major antigen	J03995.1	391/403	97
*Onchocerca flexuosa*	OFAA-aaa63a08.b1	FF141447.1	354/371	95.4
*Brugia malayi*	SW3ICA984SK	N43571.1	353/415	85.1

The BLASTp analysis of *Ol*-MJA identified MJA-like homologous sequences in 23 nematodes, showing a percentage of identical aa residues ranging from 40 to 96%, whereas the percentage of similar aa residues ranging from 47 to 98% (S3 Table). Three sequences with a percentage of identical residues between 22 and 23 aa were maintained as outgroups, with *C*. *elegans* protein annotated as rootletin. An extract of 36 full-length aa Paramyosin homologous sequences sampled among nematode and metazoan, showed a percentage of identical residues ranging from 40 to 96%, whereas the percentage of similar residues ranges from 47 to 98% (S4 Table). None of the identified sequences matched to host proteins retrievable from *Canis lupus familiaris* and other screened organisms as fungi, plants and bacteria.

**Fig 4:**
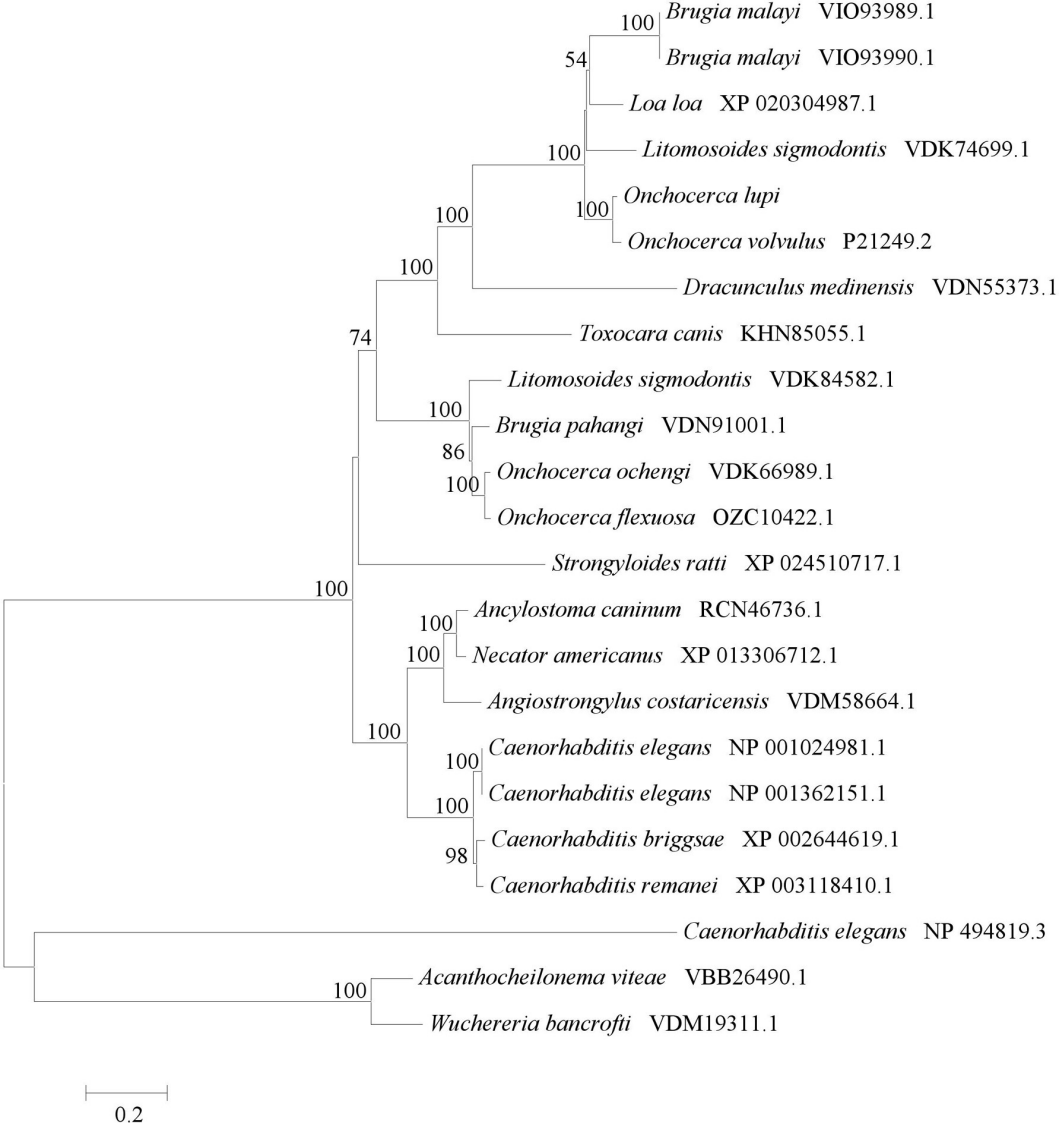
Phylogenetic analysis. Phylogenetic tree of Major antigen amino acid sequence of *Onchocerca lupi* and MJA homologous proteins of filarioid and non filarioid worms.

The phylogenetic analysis of MJA-like sampled proteins, show two main clusters grouping MJA-homologous proteins of filaroids and non-filarial worms, respectively (Fig 4). *Caenorhabditis elegans* rootletin and putative orthologs from *W*. *bancrofti* and *A*. *viteae* form a separated cluster (Fig 4).

Nucleotide and amino acid sequences of Major antigen of *O*. *lupi* were deposited in GenBank database (Accession Number MW291130).

### Prediction of signal peptides and protein 3D comparative modelling

Similarly to the results obtained from bioinformatic analyses of *Ol*-PARA [27], *Ol*-MJA sequence did not reveal signal peptide and transmembrane domains. Prosite database scan revealed the presence of several myristoylation, phosphorylation and N-linked glycosylation sites and several domain motifs, with two leucine zipper (356–377 and 1779–1800) and one RGD domains (679–681) identified.

**Fig 5:**
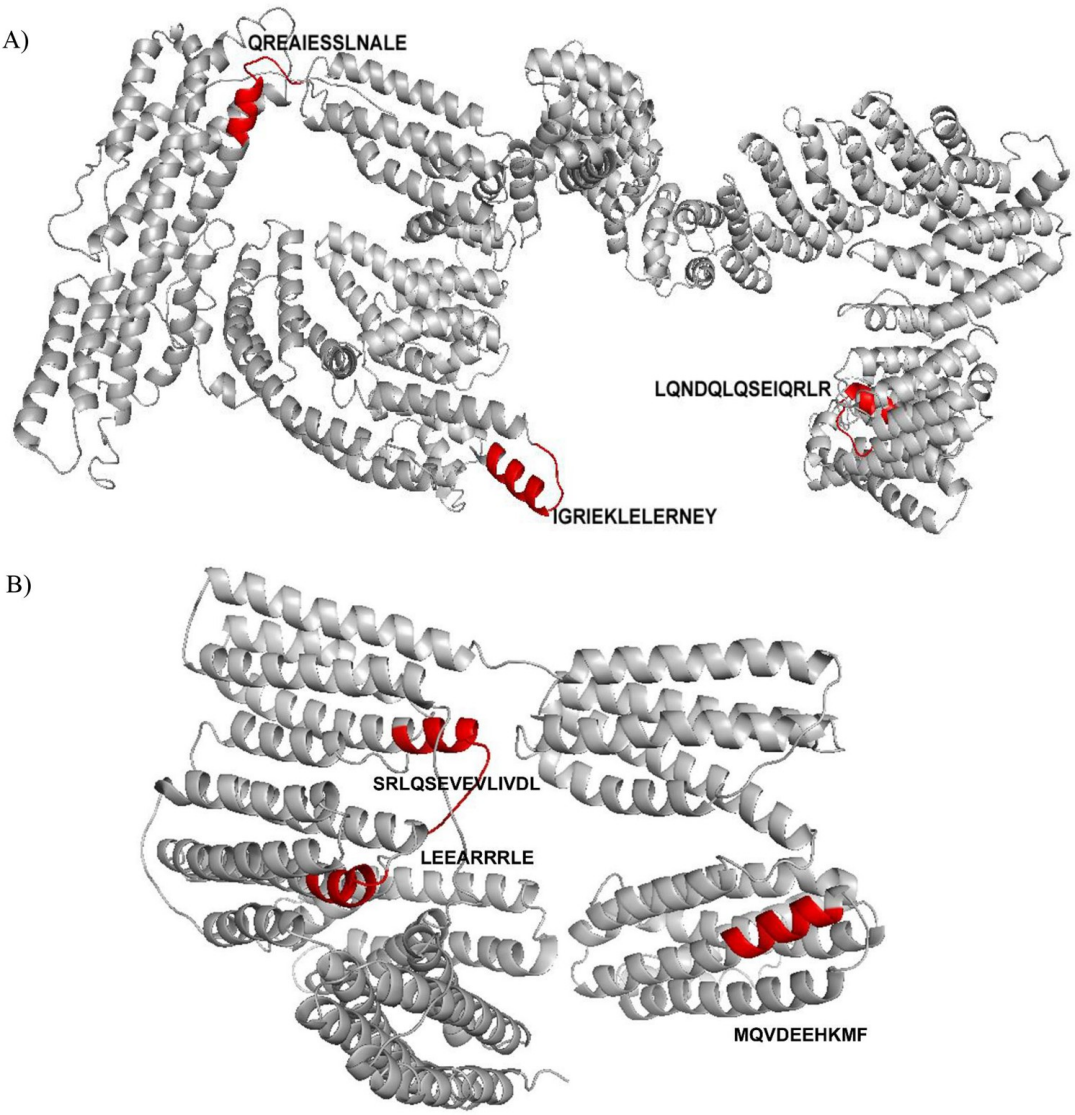
3D comparative models of *Onchocerca lupi* Major antigen (*Ol*-MJA) and *Onchocerca lupi* Paramyosin (*Ol*-PARA) proteins are reported in white cartoon representation. Red cartoon indicates the position of the three antigenic peptides identified for *Ol*-MJA (A) and *Ol*-PARA (B), respectively.

The generated multi-template 3D comparative model of *Ol*-MJA was obtained based on several crystallized structural proteins, i.e. cytoskeletal proteins (1st6.pdb), alpha actinin (1hci.pdb), tropomyosin (2tma.pdb), portion of αβ-tubulin protein heterodimers (5o09.pdb), *Saccharomyces cerevisiae* ribosome maturation factor Rea1 (6hyd.pdb) and a serine/ threonine protein kinases involved in DNA damage sensing (5yz0.pdb). Similarly, the Paramyosin 3D model was obtained based on the crystallized integrin-activating and tension-sensing focal adhesion component Talin (6r9t.pdb), the dynein-2 complex (6rlb.pdb), a SMC multi subunit complex (5xg2.pdb), the ROD domain of alpha actinin (1hci.pdb) and the microtubule associated protein PRC1 (Protein Regulator of Cytokinesis 1; 4l6y.pdb) (Fig 5A and 5B). The resulting 3D models were enriched in αhelix structures and showed the three antigenic peptides microarray mapped of *Ol*-MJA and *Ol*-PARA exposed on the proteins surface (Fig 5A and 5B).

### Linear epitope mapping and antigen selection

**Fig 6:**
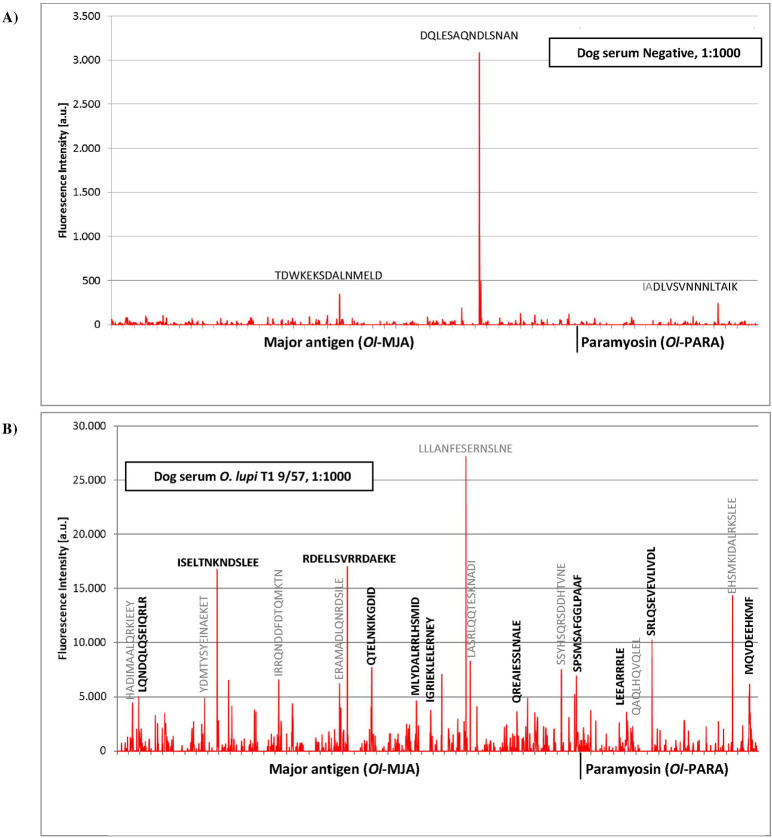
Antigen identification using high density peptide microarray against sera from uninfected and infected dogs. Intensity map of the linear peptides of *Onchocerca lupi* Major antigen (*Ol*-MJA) and *Onchocerca lupi* Paramyosin (*Ol*-PARA) probed with serum of pathogen-free dog (A). Intensity map of the linear peptides of *Ol*-MJA and *Ol*-PARA probed with *O*. *lupi* infected dog serum (ID sample: T1 9/57). In bold the linear peptides with the typical IgG response (B).

**Fig 7:**
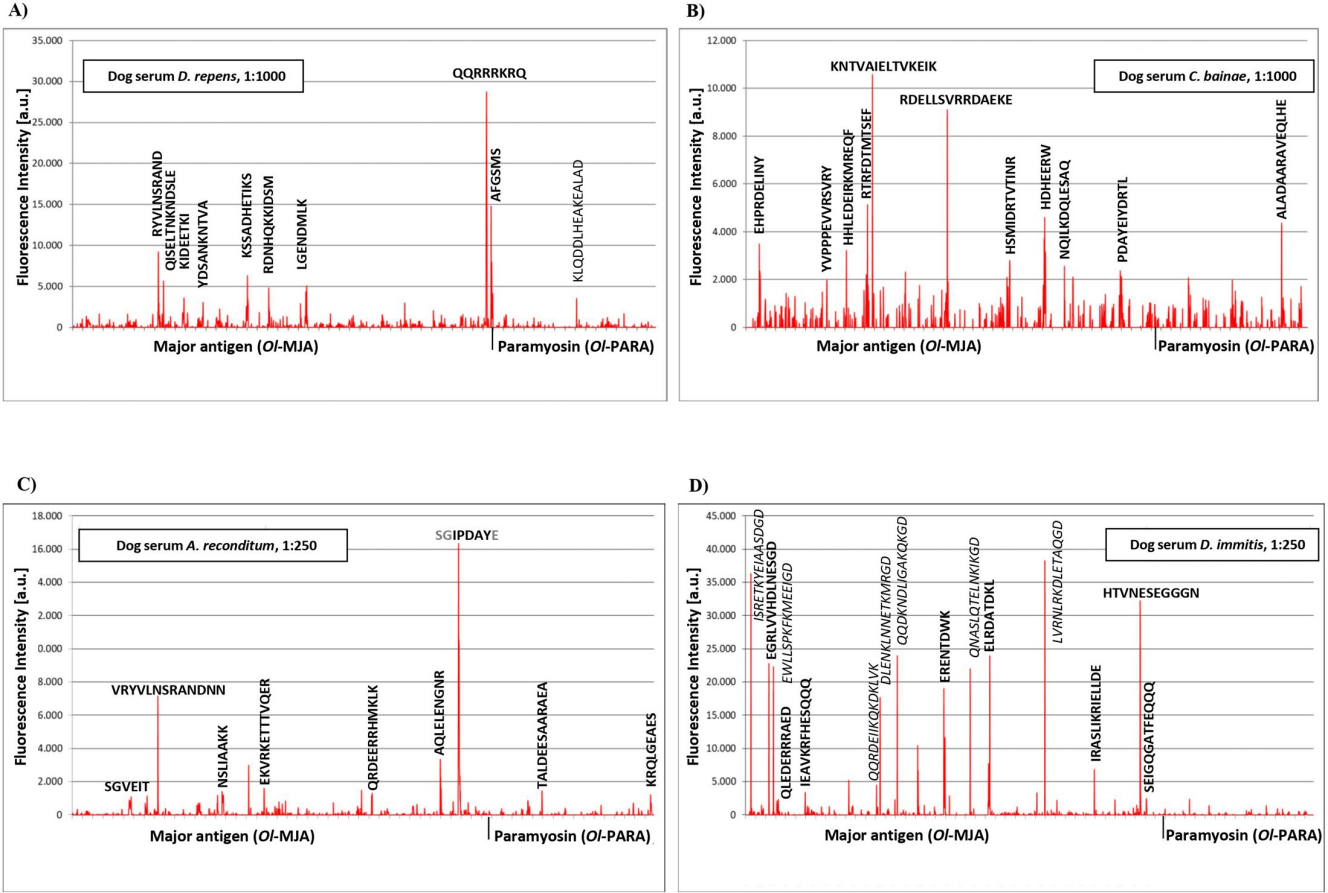
Antigen identification using high density peptide microarray against sera from infected dogs for *Dirofilaria immitis*, *Dirofilaria repens*, *Acanthocheilonema reconditum* and *Cercopithifilaria bainae*. Intensity map of the linear peptides of *Onchocerca lupi* Major antigen (*Ol*-MJA) and *Onchocerca lupi* Paramyosin (*Ol*-PARA) probed with *D*. *repens* (A), *C*. *bainae* (B), *A*. *reconditum* (C) and *D*. *immitis* (D) infected dog sera. In bold the linear peptides with the typical IgG response.

The incubation of the peptide microarray of *Ol*-MJA and *Ol*-PARA proteins with the pathogen-free dog serum resulted in a weak antibody response against the linear peptide sequences (i.e., TDWKEKSDALNMELD, DQLESAQNDLSNAN for *Ol*-MJA and IADLVSVNNNLTAIK for *Ol*-PARA) (Fig 6A and S5 Table). At a scanning intensity of 7 (red) and upon significant increase of brightness and contrast was observed a very weak background interactions of the secondary antibody with basic peptides like NIMRDQLNSERRRRR and QGATFEQQQQRRRKR presumably due to non-specific ionic binding (S5 Table). Incubation of a peptide microarray with serum of *O*. *lupi* infected dog resulted in a moderate to strong and with moderate to high spot intensities of complex IgG recognition profile against peptides of both proteins (Fig 6B and S6 Table). Some of the strongest responses were originated from single peptides-like for both *Ol*-MJA and *Ol*-PARA. Since typical epitopes exhibit lengths from 4 to 8 aa, such single peptide interactions were rather atypical and resulted from less specific interactions (Table 3). A number of epitope-like spot patterns with a clear spot morphology based on peptides with the consensus motifs were identified for *Ol*-MJA and for *Ol*-PARA proteins, respectively (Fig 6B and Tables 3 and S6). Interference with the background interactions of the secondary antibody or the antibody responses of pathogen-free dog serum was not observed. The incubation of the peptide microarray with positive dog serum for *D*. *repens* and for *C*. *bainae* showed, respectively, a complex IgG response profile with moderate to high and low to moderate spot intensities against epitope-like spot patterns formed with the consensus motifs for *Ol*-MJA and for *Ol*-PARA proteins (Fig 7A, 7B and Tables 3, S7 and S8). A single peptide (i.e., KLQDDLHEAKEALAD) for *Ol*-PARA at moderate to high signal-to-noise ratios was identified for *D*. *repens* dog positive serum (Fig 7A). Dog serum positive for *A*. *reconditum* exhibited from low to high spot intensities of IgG recognition profile against peptides of *Ol*-MJA and of *Ol*-PARA proteins (Fig 7C and Tables 3 and S9). Differently, dog serum positive for *D*. *immitis* showed a complex IgG recognition profile with high spot intensities adjacent peptides with the consensus motifs for *Ol*-MJA, but no any remarkable response against *Ol*-PARA (Fig 7D and Tables 3 and S10). Comparing the peptides identified with sera collected from *O*. *lupi* and from filarial infected dogs, six antigenic peptides (n = 3 for *Ol*-MJA; LQNDQLQSEIQRLR, IGRIEKLELERNEY, QREAIESSLNALE; and for *Ol*-PARA; LEEARRRLE, SRLQSEVEVLIVDL, MQVDEEHKMF; respectively) were selected as potential candidate biomarkers for the diagnosis of canine *O*. *lupi* infection (Tables 3 and 4).

**Table 3:** Linear antigenic peptides discovered by high-density microarray for *Onchocerca lupi* Major antigen (*Ol*-MJA) and *Onchocerca lupi* Paramyosin (*Ol*-PARA) proteins using *Onchocerca lupi*, *Dirofilaria repens*, *Cercophitifilaria bainae*, *Acanthocheilonema reconditum* and *Dirofilaria immitis* infected dog serum samples.

Protein	Species/Linear antigenic peptides				
**Major antigen**	***O*. *lupi***	***D*. *repens***	***C*. *bainae***	***A*. *reconditum***	***D*. *immitis***
	**LQNDQLQSEIQRLR**	RYVLNSRAND	EHPRDELINY	SGVEIT	ERENTDWK
	*ISELTNKNDSLEE*	QISELTNKNDSLE	YVPPPEVVRSVRY	VRYVLNSRANDNN	ELRDATDKL
	*RDELLSVRRDAEKE*	KIDEETKI	PDAYEIYDRTL	NSLIAAKK	IRASLIKRIELLDE
	*QTELNKIKGDID*	YDSANKNTVA	HHLEDEIRKMREQF	EKVRKETTTVQER	HTVNESEGGGN
	*MLYDALRRLHSMID*	KSSADHETIKS	RTRFDTMTSEF	QRDEERRHMKLK	SEIGQGATFEQQQ
	**IGRIEKLELERNEY**	RDNHQKKIDSM	KNTVAIELTVKEIK	AQLELENGNR	ISRETKYEIAASDGD*
	**QREAIESSLNALE**	LGENDMLK	RDELLSVRRDAEKE	SGIPDAYE	QNASLQTELNKIKGD*
	LLLANFESERNSLNE*	QQRRRKRQ	HSMIDRTVTINR		LVRNLRKDLETAQGD*
	SSYHSQRSDDHTVNE*		HDHEERW		QQRDEIIKQKDKLVK*
	LASRLQQTESKNADI*		NQILKDQLESAQ		IEAVKRFHESQQQ
	IRRQNDDFDTQMKTN*				QLEDERRRAED
	HADIMAALQRKIEEY*				EGRLVVHDLNESGD
	YDMTYSYEINAEKET*				QQDKNDLIGAKQKGD*
	ERAMADLQNRDSILE*				EWLLSPKFKMEEIGD*
					DLENKLNNETKMRGD*
**Paramyosin**	*SPSMSAFGGLPAAF*	AFGSMS	ALADAARAVEQLHE	TALDEESAARAEA	
	**LEEARRRLE**	KLQDDLHEAKEALAD*		KRQLGEAES	
	**SRLQSEVEVLIVDL**				
	**MQVDEEHKMF**				
	QAQLHQVQLEL*				
	EHSMKIDALRKSLEE*				

* = atypical IgG response recognition due to single peptide interaction which may result from less specific interactions or cross-reactions. Peptide sequence in italics are overlapping with antigens recognized by sera from dogs infected with other filarioids. Peptide sequences in bold are reactive uniquely to serum sample from dog infected with *Onchocerca lupi*.

**Table 4:** Intensity of reactivity, expressed as fluorescence units, of linear antigenic peptides selected for Major antigen (*Ol*-MJA) and Paramyosin (*Ol*-PARA) proteins obtained by testing *Onchocerca lupi*, *Dirofilaria repens*, *Dirofilaria immitis*, *Acanthocheilonema reconditum* and *Cercophitifilaria bainae* serum samples.

Proteins	Species					
**Major antigen**	**Peptides**	***O*. *lupi***	***D*. *repens***	***D*. *immitis***	***A*. *reconditum***	***C*. *bainae***
	LQNDQLQSEIQRLR	2.526,5	948	180,25	0	0
	IGRIEKLELERNEY	3.738	18,8	300,5	245,5	435
	QREAIESSLNALE	3.556	0	44.5	19	22
**Paramyosin**	LEEARRRLE	3.585	30	128,75	52	137,5
	SRLQSEVEVLIVDL	10.231	4	274	35,5	38,5
	MQVDEEHKMF	6.154	660,25	656,75	26,8	760,5

The BepiPred-2.0 algorithm (http://www.cbs.dtu.dk) to predict B-cell epitopes showed that almost the entire protein of *Ol*-MJA, including the peptide sequences identified experimentally, were found at 0.5 epitope threshold. Using 0.6 as epitope threshold, the AYGGGNHTSDTAITAPTGSSSYHSQRS peptide was identified. Part of this epitope- SSYHSQRSDDHTVNE—was identified to have an intensity of 7529 fluorescence units in the *O*. *lupi* positive serum and 6.5 in the uninfected dog serum confirming its potential as immunogenic epitope.

**Fig 8:**
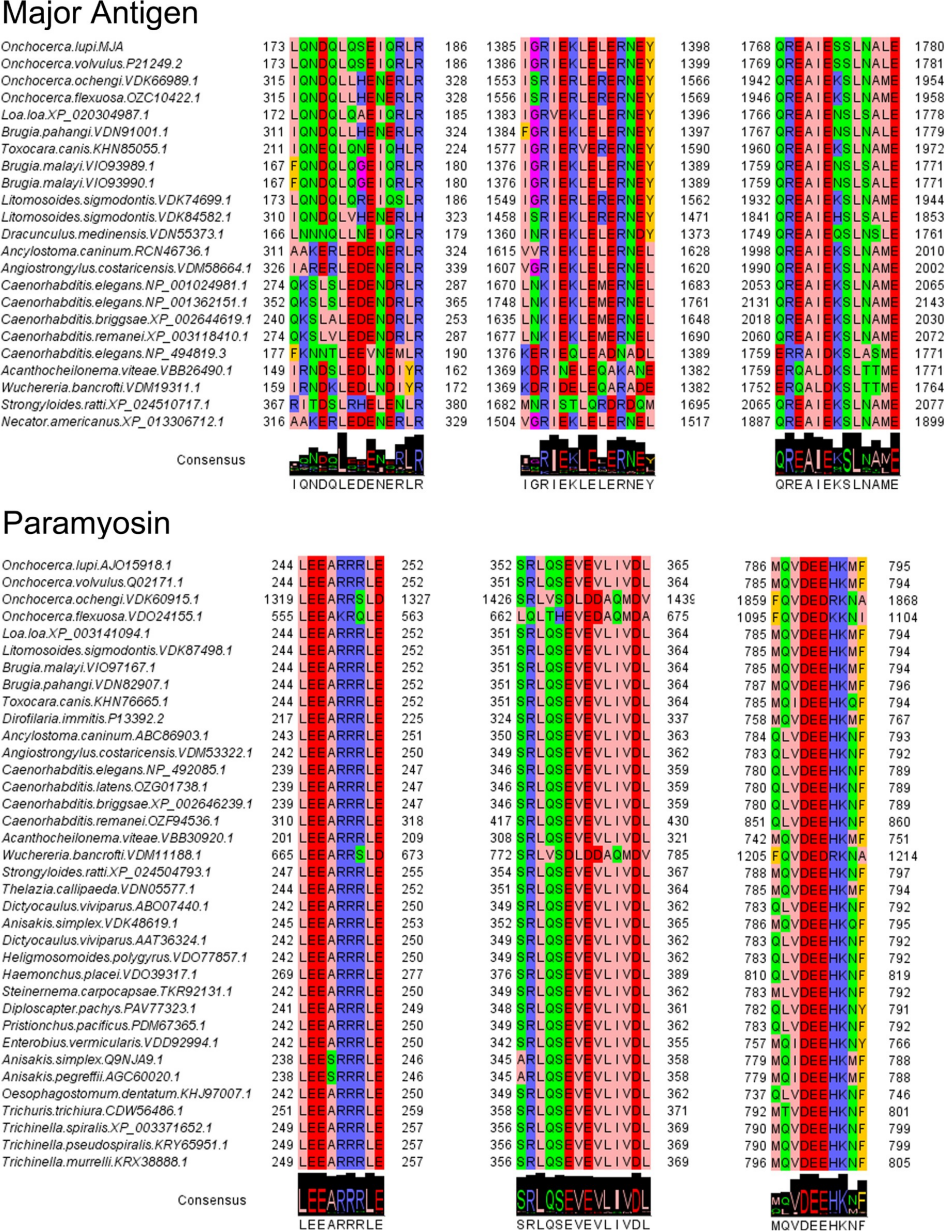
Alignment of peptides of *Onchocerca lupi* Major antigen (*Ol*-MJA) and *Onchocerca lupi* Paramyosin (*Ol*-PARA) identified with those of other nematodes. Logo representation is reported for highlighting the conserved residues.

The MSA of peptides implemented in the sequence editor package Jalview revealed that the three antigenic peptides of *Ol*-MJA (LQNDQLQSEIQRLR, IGRIEKLELERNEY, QREAIESSLNALE) showed a percentage of aa identity from 32 to 100%, from 28 to 100% and from 38 to 100% with those of other nematodes, respectively. A high value of identity (100%) was observed with those of *O*. *volvulus* (Fig 8). Differently, the antigenic peptides of *Ol*-PARA (LEEARRRLE, SRLQSEVEVLIVDL, MQVDEEHKMF) showed the aa identity ranging from 77 to 100%, from 36 to 100% and from 60 to 100%, respectively, with the high value retrieved with more than one nematodes examined (Fig 8).

## Discussion

Reactive linear peptides of *Ol-*MJA and *Ol*-PARA proteins of *O*. *lupi* were identified by means of a comprehensive strategy from sequence analyses to immunoproteomic and peptide microarray-based epitope mapping. The recognition of *Ol-*MJA and *Ol*-PARA proteins by IgG antibodies of dog infected with *O*. *lupi* indicates their appropriateness as antigen candidates to develop serological diagnostics. In addition, the molecular and proteomic dataset herein obtained for *O*. *lupi* provide firstly genetic and immunological information on this helminth. Indeed, data on *Ol*-Mja cDNA (6,163bp) and on the predicted *Ol-*MJA protein (2021 aa, 237,342 kDa), represent a unique database for this filarioid. The sequence length obtained from *Ol*-Mja cDNA was consistent with those of other nematodes such as *L*. *loa* (6,085bp), *D*. *immitis* (6,051bp) and *O*. *volvulus* (6,068bp). Analogously, the high nucleotide identity level of *Ol*-Mja with sequences from other filarioid analysed (ranging from 86.24% to 96.47%) reflects their close relationship as confirmed also by southern blot for filarial such as *Onchocerca lienalis*, *A*. *viteae*, *Brugia malayi* and *Brugia pahangi* and in none of the non-filarial species [51].

Although no data is currently available on functional aspects of *Ol*-MJA, the close phylogenetic relationship between amino acid sequences of *O*. *lupi* and *O*. *volvulus* (96.1% identity) may be of relevance for investigating analogies within the two taxa. For example, the presence of the transcript encoding for *Ol*-MJA only in adult and microfilariae stages of *O*. *lupi*, but not in eggs, was supported by studies on the *O*. *volvulus* embryogenesis [51] being *Ov*-OVT1 (234kDa) transcribed during embryonic development, only in the larval and adult forms of this parasite [51].

Similar to *Ov*-OVT1, the role of *Ol*-MJA as an embryogenesis-related protein, was supported by the detection of two leucine zipper and RGD domains as well as conserved domains as Rootletin (76–256, pfam15035) required for centrosome cohesion [52] and two SMC supefamily domains (482–1311, cl34174 and 1007–1787, cl37069) involved in the structural maintenance of chromosomes. The presence of these domains is in accordance with the function of the Ce-LFI-1 protein of *C*. *elegans* (Lin-5 (Five) Interacting protein), orthologue of *Ov*-OVT1 and *Ol*-MJA, that interacts with LIN-5 and is involved spindle positioning and chromosome segregation [53]. Furthermore, the immunofluorescence staining of *O*. *volvulus* adult worms using major antigen antisera revealed that *Ov*-OVT1 was located in the muscle of female and male parasites [54] as well as southern blot analysis recognized this protein also in the muscle of other nematodes as *B*. *malayi*, *D*. *immitis* and *C*. *elegans* [54]. Thus, *Ol*-MJA protein may have a similar muscle localization in the *O*. *lupi* nematode.

In addition, the occurrence in *Ol*-MJA of the RGD domain (detected in *Ov*-OVT1 extracellular matrix protein) [51] suggests the same role in *O*. *lupi*. However, no signal peptide as well as no transmembrane domains were found in *Ol*-MJA, therefore suggesting that an extracellular release through non-canonical pathway might occur. Therefore, considering that antigenic proteins should be located on the plasma membrane or secreted, *Ol*-MJA protein can exert its immunological function in other ways still unknown. Indeed, the secreted proteins are likely to represent the principal immunologically active products exerting an effect on the host system [55]. Overall, it is known that the infection by helminth parasites, including *Onchocerca* spp., strongly stimulate the hosts immune response [56]. In this context, the potential immunological role of *Ol*-MJA protein, may be assumed considering the immunogenicity properties associated to *Ov*-OVT1, as previously demonstrated by stimulation of the antibodies production in rabbits [51]. Furthermore, the exposure of the epitopes observed in *Ol*-MJA 3D structure may support the immunological potential of this protein. In addition, the immunogenicity of *Ol*-MJA may be also reinforced by the partial 3D structure homology with those of other myosin-like proteins (i.e., tropomyosin, myosin, actin), as previously described for *Schistosoma mansoni* [57] and for *Fasciola hepatica*, the latter conferring protection in *F*. *hepatica* experimentally infected rats [58].

The main concerns of studies that involve serological assessments are the sensitivity and cross-reactivity amongst antibodies and antigens of related pathogens as previously reported [24, 59]. Indeed, several studies have been attempted to adapt existing diagnostic methods for the detection of filaroids taking advantage of the cross-reactions between antibodies and similar antigens. For example, the Og4C3 monoclonal antibodies initially developed against non-phosphorylcholine antigens of *Onchocerca gibsoni* showed to cross-react with antigens of *W*. *bancrofti* [59, 60]. Similarly, the ELISA kit using Og4C3 antibodies tested with sera of *O*. *lupi*-infected dogs showed a low sensitivity detecting only three out of six infected serum samples [26].

Although, this study has some limitations (i.e., data were obtained from a single serum sample from each infected dog and *Ol*-MJA and *Ol*-PARA were examined as short peptides which may not include all the immunoreactive epitopes), the epitope microarray assay demonstrated a broad IgG immune response directed against three linear epitopes for *Ol*-MJA (LQNDQLQSEIQRLR, IGRIEKLELERNEY, QREAIESSLNALE). Nevertheless, their specificity and reactivities as well as the conformational structure need to be validated with a larger number of serum samples, considering that the immune response to parasites may vary significantly from one individual to another. However, the immunological potential of linear antigenic epitopes has been demonstrated by microarray screening of the entire proteome of different pathogens [61–63], as it is the case of *O*. *volvulus* for which three peptide motifs with an IgG1, IgG3, IgE and IgM response profile were identified [64]. Furthermore, though Paramyosin is a widely conserved protein across species with well-known antigenic potential [65, 66], the immunological properties of *Ol*-PARA [25] have been herein investigated by the identification of the three linear epitopes (LEEARRRLE, SRLQSEVEVLIVDL, MQVDEEHKMF), which showed a high intensity of reactivity when tested against serum sample of *O*. *lupi* infected dog.

Even if the high epitopes identity were observed for both proteins when compared with those of other nematodes, the results obtained both *in silico* and by the peptide microarray assay may potentially lead to the development of novel serological tests useful for diagnostic and immunoprophylactic studies for canine onchocercosis. However, their sensitivity and specificity need to be tested in a larger cohort of *O*. *lupi*-infected dogs coming from different geographical areas as well as further evaluation of co-infections, also considering the individual variation in reactivity patterns of each dog. Moreover, the comparison of the performances of these biomarkers in latent and patent phases is another important aspect that needs to be evaluated together with detection in subclinical or asymptomatic cases.

## Conclusions

The development of an adequate diagnostic tool will enable further studies on the distribution and prevalence of this little known zoonotic filarioid. Although, other investigations are necessary to determine the utility of the epitopes identified (alone or in combination) for the diagnosis of canine onchocercosis, for mapping or monitoring the diseases, without excluding the possibility of its application even in the diagnosis of feline and human infection, this study indicates that linear peptides of *Ol*-MJA and *Ol*-PARA proteins may be suitable candidates for the development of a diagnostic test.

## Supporting information

S1 Fig SDS-PAGE. Lines 1–8: *Onchocerca lupi* female protein extract in reducing conditions. L: Ladder.
(TIF)

S1 Table Primers designed for *Onchocerca lupi* Major antigen amplification and PCR run protocols. (DOC)

S2 Table Proteins and peptides reactive to serum from *Onchocerca lupi*-infected dog identified in the two gel bands (lower and upper) by nLC-MS/MS analysis. The SDS-PAGE bands at 100kDa (lower) and 200kDa (upper), reactive to serum from *O*. *lupi*-infected dog, were excised and submitted to in-gel digestion procedure. The resulting peptides were analysed by nLC-MS/MS and identified by database searched. The protein name, ID, peptide sequence and Mascot ion scores are reported. Peptides assigned to *Onchocerca volvulus* proteins are coloured.
(XLS)

S3 Table MatGAT2.01 analyses of *Ol*-MJA homologous sequences sampled by BLASTp through nematodes. (XLS)

S4 Table MatGAT2.01 analyses of *Ol*-PARA homologous sequences sampled by BLASTp through nematodes. (XLS)

S5 Table High density peptide array screening using the serum from dog infected with *Onchocerca lupi*. *Ol*-MJA and *Ol*-PARA sequences were screened scanning the entire sequence using a 15mer peptide. The parameters included in the table are: the row and column number of the microarray plate, peptide sequence, signal intensity against *O*. *lupi* dog serum diluted 1:1000, standard deviation, deviation [%], corrected signal intensity against *O*. *lupi* dog serum diluted 1:1000. The table was generated from the formatted raw data and lists averaged spot intensities and spot-to-spot deviations. Spots with a deviation of >40% were zeroed in the column with the corrected intensity values.
(XLSX)

S6 Table High density peptide array screening using the serum from pathogen-free dog. *Ol*-MJA and *Ol*-PARA sequences were screened scanning the entire sequence using a 15mer peptide. The parameters included in the table are: the row and column number of the microarray plate, peptide sequence, signal intensity against *O*. *lupi* dog serum diluted 1:1000, standard deviation, deviation [%], corrected signal intensity against *O*. *lupi* dog serum diluted 1:1000. The table was generated from the formatted raw data and lists averaged spot intensities and spot-to-spot deviations. Spots with a deviation of >40% were zeroed in the column with the corrected intensity values.
(XLSX)

S7 Table High density peptide array screening using the serum from dog infected with *Dirofilaria repens*. *Ol*-MJA and *Ol*-PARA sequences were screened scanning the entire sequence using a 15mer peptide. The parameters included in the table are: the row and column number of the microarray plate, peptide sequence, signal intensity against *D*. *repens* dog serum diluted 1:1000, standard deviation, deviation [%], corrected signal intensity against *D*. *repens* dog serum diluted 1:1000. The table was generated from the formatted raw data and lists averaged spot intensities and spot-to-spot deviations. Spots with a deviation of >40% were zeroed in the column with the corrected intensity values.
(XLSX)

S8 Table High density peptide array screening using the serum from dog infected with *Cercopithifilaria bainae*. *Ol*-MJA and *Ol*-PARA sequences were screened scanning the entire sequence using a 15mer peptide. The parameters included in the table are: the row and column number of the microarray plate, peptide sequence, signal intensity against *C*. *bainae* dog serum diluted 1:1000, standard deviation, deviation [%], corrected signal intensity against *C*. *bainae* dog serum diluted 1:1000. The table was generated from the formatted raw data and lists averaged spot intensities and spot-to-spot deviations. Spots with a deviation of >40% were zeroed in the column with the corrected intensity values.
(XLSX)

S9 Table High density peptide array screening using the serum from dog infected with *Acanthocheilonema reconditum*. *Ol*-MJA and *Ol*-PARA sequences were screened scanning the entire sequence using a 15mer peptide. The parameters included in the table are: the row and column number of the microarray plate, peptide sequence, signal intensity against *A*. *reconditum* dog serum diluted 1:250, standard deviation, deviation [%], corrected signal intensity against *A*. *reconditum* dog serum diluted 1:250. The table was generated from the formatted raw data and lists averaged spot intensities and spot-to-spot deviations. Spots with a deviation of >40% were zeroed in the column with the corrected intensity values.
(XLSX)

S10 Table High density peptide array screening using the serum from dog infected with *Dirofilaria immitis*. *Ol*-MJA and *Ol*-PARA sequences were screened scanning the entire sequence using a 15mer peptide. The parameters included in the table are: the row and column number of the microarray plate, peptide sequence, signal intensity against *D*. *immitis* dog serum diluted 1:250, standard deviation, deviation [%], corrected signal intensity against *D*. *immitis* dog serum diluted 1:250. The table was generated from the formatted raw data and lists averaged spot intensities and spot-to-spot deviations. Spots with a deviation of >40% were zeroed in the column with the corrected intensity values.
(XLSX)

## References

[1] Lefoulon E, Giannelli A, Makepeace BL, Mutafchiev Y, Townson S, Uni S, et al Whence river blindness? The domestication of mammals and host-parasite co-evolution in the nematode genus *Onchocerca*. Int J Parasitol. 2017;47: 457–470. 10.1016/j.ijpara.2016.12.009 28344097

[2] Eberhard ML, Ortega Y, Dial S, Schiller CA, Sears AW, Greiner E. Ocular *Onchocerca* infections in two dogs in western United States. Vet Parasitol. 2000;90: 333–338. 10.1016/s0304-4017(00)00252-1 10856819

[3] Bain O. Evolutionary relationships among filarial nematodes Klei TR, Rajan TV, editors. The Filaria. Boston, USA: Kluwer Academic Publishers; 2002 pp. 21–29.

[4] Borup LH, Peters JS, Sartori CR. Onchocerciasis (river blindness). Cutis. 2003; 72: 297–302. 14604081

[5] Sreter T, Szell Z, Egyed Z, Varga I. Subconjunctival zoonotic onchocerciasis in man: aberrant infection with *Onchocerca lupi*? Ann Trop Med Parasitol. 2002;96: 497–502. 10.1179/000349802125001267 12194710

[6] Sreter T, Szell Z. Onchocercosis: a newly recognized disease in dogs. Vet Parasitol 2008;15: 1–13. 10.1016/j.vetpar.2007.09.008 17951007

[7] Otranto D, Sakru N, Testini G, Gürlü VP, Yakar K, Lia RP, et al Case report: First evidence of human zoonotic infection by *Onchocerca lupi* (Spirurida, Onchocercidae). Am J Trop Med Hyg. 2011;84: 55–58. 10.4269/ajtmh.2011.10-0465 21212202 PMC3005520

[8] Otranto D, Dantas-Torres F, Giannelli A, Abramo F, Ignjatović Ćupina A, Petrić D, et al Cutaneous distribution and circadian rhythm of *Onchocerca lupi* microfilariae in dogs. PLoS Negl Trop Dis. 2013;7: e2585 10.1371/journal.pntd.0002585 24349594 PMC3861181

[9] Otranto D, Giannelli A, Trumble SN, Chavkin M, Kennard G, Latrofa MS, et al Clinical case presentation and a review of the literature of canine onchocercosis by *Onchocerca lupi* in the United States. Parasit Vectors. 2015;8: 89–96. 10.1186/s13071-015-0699-3 25884672 PMC4346121

[10] Rodonaja TE. A new species of nematode, *Onchocerca lupi* n. sp., from *Canis lupus cubanensis*. Bulletin of the Academic of Science of Georgian SSR. 1967;45: 715–719.

[11] Hermosilla C, Hetzel U, Bausch M, Grübl J, Bauer C. First autochthonous case of canine ocular onchocercosis in Germany. Vet Rec. 2005;156: 450–452. 10.1136/vr.156.14.450 15828728

[12] Szell Z, Erdelyi I, Sreter T, Albert M, Varga I. Canine ocular onchocercosis in Hungary. Vet Parasitol. 2001;97: 243–249. 10.1016/s0304-4017(01)00397-1 11390077

[13] Labelle AL, Daniels JB, Dix M, Labelle P. *Onchocerca lupi* causing ocular disease in two cats. Vet Ophthalmol. 2011;14: 105–110. 10.1111/j.1463-5224.2011.00911.x 21923832

[14] Labelle AL, Maddox CW, Daniels JB, Lanka S, Eggett TE, Dubielzig RR, et al Canine ocular onchocercosis in the United States is associated with *Onchocerca lupi*. Vet Parasitol. 2013;193: 297–301. 10.1016/j.vetpar.2012.12.002 23276598

[15] Maia C, Annoscia G, Latrofa MS, Pereira A, Giannelli A, Pedroso L, et al *Onchocerca lupi* nematode in cat, Portugal. Emerg Infect Dis. 2015;21: 2252–2253. 10.3201/eid2112.150061 26584050 PMC4672443

[16] Otranto D, Dantas-Torres F, Giannelli A, Latrofa MS, Papadopoulos E, Cardoso L, et al Zoonotic *Onchocerca lupi* in dogs from Greece and Portugal. Emerg Infect Dis. 2013;9: 2000–2003. 10.3201/eid1912.130264 24274145 PMC3840859

[17] Miró G, Montoya A, Checa R, Gálvez R, Mínguez JJ, Marino V, et al First detection of *Onchocerca lupi* infection in dogs in southern Spain. Parasit Vectors. 2016;9: 290–292. 10.1186/s13071-016-1587-1 27193758 PMC4872350

[18] Berry R, Stepenaske S, DehorityW, Almeida M, Nmathison B, Bishop H, et al. Zoonotic *Onchocerca lupi* presenting as a subcutaneous nodule in a 10-year-old girl: report of the second case in the United states and a review of the literature. Abstract Book of ASDP annual meeting, Nov 6–9, 2014, Chicago, USA.

[19] Dudley RW, Smith C, Dishop M, Mirsky D, Handler MH, Rao S. A cervical spine mass caused by *Onchocerca lupi*. Lancet. 2015;386: 1372 10.1016/S0140-6736(14)62255-8 25843892

[20] Grácio AJ, Richter J, Komnenou AT, Grácio MA. Onchocerciasis caused by *Onchocerca lupi*: an emerging zoonotic infection. Systematic review. Parasitol Res. 2015;114: 2401–2413. 10.1007/s00436-015-4535-7 25990062

[21] Egyed Z, Sréter T, Széll Z, Beszteri B, Oravecz O, Marialigeti K, et al Morphologic and genetic characterization of *Onchocerca lupi* infecting dogs. Vet Parasitol. 2001;102: 309–319. 10.1016/s0304-4017(01)00541-6 11731074

[22] Sréter-Lancz Z, Széll Z, Sréter T. Molecular genetic comparison of *Onchocerca* sp. Infecting dogs in Europe with other spirurid nematodes including *Onchocerca lienalis*. Vet Parasitol. 2007;148: 365–370. 10.1016/j.vetpar.2007.06.021 17673369

[23] Zarfoss MK, Dubielzig RR, Eberhard ML, Schmidt KS. Canine ocular onchocerciasis in the United States: two new cases and a review of the literature. Vet Ophthalmol. 2005;8: 51–57. 10.1111/j.1463-5224.2005.00348.x 15644101

[24] Komnenou AT, Thomas ALN, Papadopoulos E, Koutinas AF. Intraocular localization of *Onchocerca lupi* adult worm in a dog with anterior uveitis: a case report. Vet Ophthalmol. 2015;19: 245–249. 10.1111/vop.12277 25929486

[25] Latrofa MS, Annoscia G, Colella V, Cavalera MA, Maia C, Martin C, et al A real-time PCR tool for the surveillance of zoonotic *Onchocerca lupi* in dogs, cats and potential vectors. PLoS Negl Trop Dis. 2018;12: e0006402 10.1371/journal.pntd.0006402 29617361 PMC5902036

[26] Giannelli A, Cantacessi C, Graves P, Becker L, Campbell BE, Dantas-Torres F et al A preliminary investigation of serological tools for the detection of *Onchocerca lupi* infection in dogs. Parasitol Res. 2014;113: 1989–1991. 10.1007/s00436-014-3844-6 24647986

[27] Campbell B, Cortes H, Annoscia G, Giannelli A, Parisi A, Latrofa MS, et al Paramyosin of canine *Onchocerca lupi*: usefulness for the diagnosis of a neglected zoonotic disease. Parasit Vectors. 2016;9: 493 10.1186/s13071-016-1783-z 27604904 PMC5013582

[28] Colella V, Maia C, Pereira A, Gonçalves N, Caruso M, Martin C, et al Evaluation of oxfendazole in the treatment of zoonotic *Onchocerca lupi* infection in dogs. PLoS Negl Trop Dis. 2018;1: e0006218 10.1371/journal.pntd.0006218 29377880 PMC5805361

[29] Otranto D, Testini G, Dantas-Torres F, Latrofa MS, Diniz PP, de Caprariis D, et al Diagnosis of canine vector-borne diseases in young dogs: a longitudinal study. J Clin Microbiol. 2010;48: 3316–3324. 10.1128/JCM.00379-10 20660218 PMC2937705

[30] Laemmli UK. Cleavage of Structural Proteins during the Assembly of the Head of Bacteriophage T4. Nature. 1970;227: 680–685. 10.1038/227680a0 5432063

[31] Renart J, Reiser J, Stark GR. Transfer of proteins from gels to diazobenzyloxymethyl-paper and detection with antisera: a method for studying antibody specificity and antigen structure. PNAS. 1979;76: 3116–3120. 10.1073/pnas.76.7.3116 91164 PMC383774

[32] Shevchenko A, Jensen ON, Podtelejnikov AV, Sagliocco F, Wilm M, Vorm O, et al Linking genome and proteome by mass spectrometry: large-scale identification of yeast proteins from two dimensional gels. Proc Natl Acad Sci U S A. 1996;93: 14440–14445. 10.1073/pnas.93.25.14440 8962070 PMC26151

[33] Rappsilber J, Ishihama Y, Mann M. Stop and go extraction tips for matrix-assisted laser desorption/ionization, nanoelectrospray, and LC/MS sample pretreatment in proteomics. Anal Chem. 2003;75: 663–670. 10.1021/ac026117i 12585499

[34] Perez-Riverol Y, Csordas A, Bai J, Bernal-Llinares M, Hewapathirana S, Kundu DJ, et al The PRIDE database and related tools and resources in 2019: improving support for quantification data. Nucleic Acids Res. 2019;47: D442–D450. 10.1093/nar/gky1106 30395289 PMC6323896

[35] Käll L, Canterbury JD, Weston J, Noble WS, MacCoss MJ. Semi-supervised learning for peptide identification from shotgun proteomics datasets. Nat Methods. 2007;4: 923–925. 10.1038/nmeth1113 17952086

[36] Keller A, Nesvizhskii AI, Kolker E, Aebersold R. Empirical statistical model to estimate the accuracy of peptide identifications made by MS/MS and database search. Anal Chem. 2002;74: 5383–5392. 10.1021/ac025747h 12403597

[37] Nesvizhskii AI, Keller A, Kolker E, Aebersold R. A statistical model for identifying proteins by tandem mass spectrometry. Anal Chem. 2003;75: 4646–4658. 10.1021/ac0341261 14632076

[38] Untergasser A, Cutcutache I, Koressaar T, Ye J, Faircloth BC, Remm M, et al Primer3—new capabilities and interfaces. Nucleic Acids Res. 2012;40: e115 10.1093/nar/gks596 22730293 PMC3424584

[39] Tamura K, Peterson D, Peterson N, Stecher G, Nei M, Kumar S. MEGA5: molecular evolutionary genetics analysis using maximumlikelihood, evolutionary distance, and maximum parsimony methods. Mol Biol Evol. 2011;28: 2731–2739. 10.1093/molbev/msr121 21546353 PMC3203626

[40] Zhang Z, Schwartz S, Wagner L, Miller W. A greedy algorithm for aligning DNA sequences. J Comput Biol. 2000;7: 203–214. 10.1089/10665270050081478 10890397

[41] Altschul SF, Gish W, Miller W, Myers EW, Lipman DJ. Basic local alignment search tool. J Mol Biol. 1990;215: 403–410. 10.1016/S0022-2836(05)80360-2 2231712

[42] Waterhouse AM, Procter JB, Martin DM, Clamp M, Barton GJ. Jalview Version 2-a multiple sequence alignment editor and analysis workbench. Bioinformatics. 2009;25: 1189–1191. 10.1093/bioinformatics/btp033 19151095 PMC2672624

[43] Jones DT, Taylor WR, Thornton JM. The rapid generation of mutation data matrices from protein sequences. Computer Applications in the Biosciences. 1992;8: 275–282. 10.1093/bioinformatics/8.3.275 1633570

[44] Kimura M. A simple method for estimating evolutionary rate of base substitutions through comparative studies of nucleotide sequences. J Mol Evol. 1980;16: 111–120. 10.1007/BF01731581 7463489

[45] Nielsen H, Krogh A. Prediction of signal peptides and signal anchors by a hidden Markov model. Proc Int Conf Intell Syst Mol Biol. 1998;6: 122–130. 9783217

[46] Bendtsen JD, Nielsen H, von Heijne G, Brunak S. Improved prediction of signal peptides: SignalP. J Mol Biol. 2004;340: 783–790. 10.1016/j.jmb.2004.05.028 15223320

[47] Petersen TN, Brunak S, von Heijne G, Nielsen H. SignalP 40: discriminating signal peptides from transmembrane regions. Nat Methods. 2011;8: 785–786. 10.1038/nmeth.1701 21959131

[48] Marchler-Bauer A, Bryant SH. CD-Search: protein domain annotations on the fly. Nucleic Acids Res. 2004;32: W327–331. 10.1093/nar/gkh454 15215404 PMC441592

[49] Bertoni M, Kiefer F, Biasini M, Bordoli L, Schwede T. Modeling protein quaternary structure of homo- and hetero-oligomers beyond binary interactions by homology. Sci Rep. 2017;7: 10480 10.1038/s41598-017-09654-8 28874689 PMC5585393

[50] Pierri CL, Parisi G, Porcelli V. Computational approaches for protein function prediction: A combined strategy from multiple sequence alignment to molecular docking-based virtual screening. Biochim Biophys Acta. 2010;1804: 1695–1712. 10.1016/j.bbapap.2010.04.008 20433957

[51] Triteeraprapab S, Richie TL, Tuan RS, Shepley KJ, Dinman JD, Neubert TA, et al Molecular cloning of a gene expressed during early embryonic development in *Onchocerca volvulus*. Mol Biochem Parasitol. 1995;69: 161–171. 10.1016/0166-6851(94)00187-r 7770081

[52] Graser S, Stierhof YD, Nigg EA. Cep68 and Cep215 (Cdk5rap2) are required for centrosome cohesion. J Cell Sci. 2007;120: 4321–4331. 10.1242/jcs.020248 18042621

[53] Fisk Green R, Lorson M, Walhout AJ, Vidal M, van den Heuvel S. Identification of critical domains and putative partners for the *Caenorhabditis elegans* spindle component LIN-5. Mol Genet Genomics. 2004;271: 532–544. 10.1007/s00438-004-1012-x 15138888

[54] Erondu NE, Donelson JE. Characterization of a myosin-like antigen from *Onchocerca volvulus*. Mol Biochem Parasitol. 1990;40: 213–224. 10.1016/0166-6851(90)90043-l 2194123

[55] Maizels RM, Gomez-Escobar N, Gregory WF, Murray J, Zang X. Immune evasion genes from filarial nematodes. Int J Parasitol. 2001;31: 889–898. 10.1016/s0020-7519(01)00213-2 11406138

[56] Maizels RM, Balic A, Gomez-Escobar N, Nair M, Taylor MD, et al Helminth parasites masters of regulation. Immunol Rev. 2004;201: 89–116. 10.1111/j.0105-2896.2004.00191.x 15361235

[57] Newport GR, Harrison RA, McKerrow J, Tarr P, Kallestad J, Agabian N. Molecular cloning of *Schistosoma mansoni* myosin. Mol Biochem Parasitol. 1987;26: 29–38. 10.1016/0166-6851(87)90127-7 3431565

[58] Henker LC, Schwertz CI, Lucca NJ, Piva MM, Prior KC, Baska P, et al Immune protection conferred by recombinant MRLC (myosin regulatory light chain) antigen in TiterMax Gold adjuvant against experimental fasciolosis in rats. Vaccine. 2017;35: 663–671. 10.1016/j.vaccine.2016.11.092 28024953

[59] More SJ, Copeman DB. A highly specific and sensitive monoclonal antibody-based ELISA for the detection of circulating antigen in bancroftian filariasis. Trop Med Parasitol. 1990;41: 403–406. 2075384

[60] Chanteau S, Moulia-Pelat JP, Glaziou P, Nguyen NL, Luquiaud P, Plichart C, et al Og4C3 circulating antigen: a marker of infection and adult worm burden in *Wuchereria bancrofti* filariasis. J Infect Dis. 1994;170: 247–250. 10.1093/infdis/170.1.247 8014511

[61] Gonzalez-Moa MJ, Van Dorst B, Lagatie O, Verheyen A, Stuyver L, Biamonte MA. Proof-of-Concept Rapid Diagnostic Test for Onchocerciasis: Exploring Peptide Biomarkers and the Use of Gold Nanoshells as Reporter Nanoparticles. ACS Infect. Dis. 2018;4,6: 912–917. 10.1021/acsinfecdis.8b00031 29547260

[62] Melnyk O, Duburcq X, Olivier C, Urbès F, Auriault C, Gras-Masse H. Peptide arrays for highly sensitive and specific antibody-binding fluorescence assays. Bioconjug Chem. 2002;13: 713–720. 10.1021/bc015584o 12121125

[63] Carmona SJ, Sartor PA, Leguizamón MS, Campetella OE, Agüero F. Diagnostic peptide discovery: prioritization of pathogen diagnostic markers using multiple features. PLoS One. 2012;7: e50748 10.1371/journal.pone.0050748 23272069 PMC3522711

[64] Lagatie O, Van Dorst B, Stuyver LJ. Identification of three immunodominant motifs with atypical isotype profile scattered over the *Onchocerca volvulus* proteome. PLoS Negl Trop Dis. 2017;11: e0005330 10.1371/journal.pntd.0005330 28125577 PMC5295699

[65] Abou-Elhakam HMA, Bauomy IR, El Deeb SO, El Amir AM. Immunodiagnosis of fasciolasis using sandwich enzyme-linked immunosorbent assay for detection of *Fasciola gigantica* paramyosin antigen. Tropical Parasitol. 2013;3: 44–52. 10.4103/2229-5070.113907 PMC3745671 23961441

[66] McManus DP, Liu S, Song G, Xu Y, Wong JM. The vaccine efficacy of native paramyosin (Sj-97) against Chinese *Schistosoma japonicum*. Int J Parasitol. 1998;28: 1739–1742. 10.1016/s0020-7519(98)00151-9 9846611

