# Supplementary figures and images for: Major antigen and paramyosin proteins as candidate biomarkers for serodiagnosis of canine infection by zoonotic Onchocerca lupi
Source: PLoS Negl Trop Dis. 2021 Feb 10;15(2):e0009027. doi: 10.1371/journal.pntd.0009027 (PMC7875354; doi:10.1371/journal.pntd.0009027)

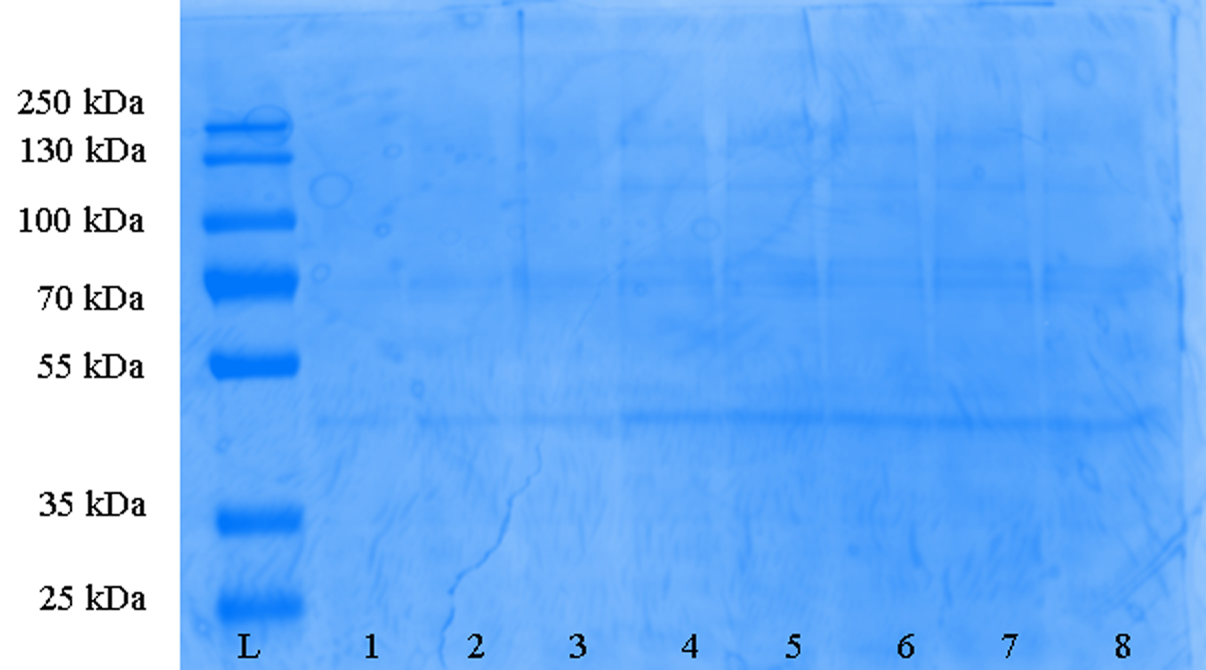

Supplement: S1 Fig — L: Ladder. (TIF) [file pntd.0009027.s001.tif]
