# Supplementary material for: Major antigen and paramyosin proteins as candidate biomarkers for serodiagnosis of canine infection by zoonotic Onchocerca lupi
Source: PLoS Negl Trop Dis. 2021 Feb 10;15(2):e0009027. doi: 10.1371/journal.pntd.0009027 (PMC7875354; doi:10.1371/journal.pntd.0009027)
Supplement: S1 Table — (DOC) [file pntd.0009027.s002.doc]

**Supplementary Table 1.** **Primers designed for *Onchocerca lupi* Major antigen amplification and PCR run protocols.**

|  | Primers sequence 5’-3’ | Fragment size (bp) | PCR protocol | | |
| --- | --- | --- | --- | --- | --- |
|  |  |  | Step | T (°C) | t |
| Fragment 1 | *O.l._*1_Mja_F-CTTCTGCCACCGCAACATTC | 840 | Denaturation | 98 | 2 ‘ |
|  | *O.l*._1_Mja_R-CTGTTTGTTCTCGATATTCGG |  | 40 Cycles | 98  60  72 | 10 “  20 “  210‘‘ |
|  |  |  | Extension | 72 | 7 ’ |
|  |  |  |  |  |  |
| Fragment 2 | *O.l._*2_Mja_F-CTTCAAAATGATCAGTTACAGAGTGAGATTCAG | 1100 | Denaturation | 98 | 2 ‘ |
|  | *O.l*._2_Mja_R-GCGTATTTTAGTTTCCTCATCGATTTT |  | 40 Cycles | 98  58  60 | 10 “  20 “  210“ |
|  |  |  | Extension | 68 | 7 ’ |
| Fragment 3 | *O.l._*3_Mja_F-AGTCTTGAACAGGAAAAG | 1350 | Denaturation | 98 | 2 ‘ |
|  | *O.l*._3_Mja_R-TTTTTCAATTTCAGTTTGTAATTT |  | 30 Cycles | 98  58  60 | 10 “  15 “  180“ |
|  |  |  | Extension | 68 | 7 ‘ |
|  |  |  |  |  |  |
| Fragment 4 | *O.l._*4_Mja_F-CGCGCGATAGCAAAAGCG | 2060 | Denaturation | 98 | 2 ‘ |
|  | *O.l*._4_Mja_R-GCGAATTTGTTGCGATTCACTTTCTTG |  | 40 Cycles | 98  58  72 | 10 “  20 “  210‘‘ |
|  |  |  | Extension | 72 | 7 ’ |
|  |  |  |  |  |  |
| Fragment 5 | *O.l._*5_Mja_F-GATCAAGAAAGTGAATCGCAACAAATTCGC | 690 | Denaturation | 98 | 2 ‘ |
|  | *O.l*._5_Mja_R-TCGTTTTTCCTCATCGAGTAATTCAATGCG |  | 30 Cycles | 98  58  60 | 10 “  15 “  180“ |
|  |  |  | Extension | 68 | 7 ‘ |
|  |  |  |  |  |  |
| Fragment 6 | *O.l._*6_Mja_F-AATACATTACAAAACGACCTCCGT | 930 | Denaturation | 98 | 2 ‘ |
|  | *O.l*._6_Mja_R-CTCTGCTGCTGTTGCTCAAATGTCGCAC |  | 40 Cycles | 98  58  60 | 10 “  20 “  210“ |
|  |  |  | Extension | 68 | 7 ‘ |
|  |  |  |  |  |  |
| Fragment 7 | *O.l._*7_Mja_F-GAAGTGACACTCATACTGTC | 300 | Denaturation | 94 | 10 ‘ |
|  | *O.l._*7_Mja_R-GAATTCCCAAAGAATCTATATC |  | 30 Cycles | 94  63  72 | 30“  30“  20“ |
|  |  |  | Extension | 72 | 5 ‘ |
